# Intra-abdominal hypertension; prevalence, incidence and outcomes in a low resource setting; a prospective observational study

**DOI:** 10.1186/s13017-015-0051-4

**Published:** 2015-11-24

**Authors:** Job Kuteesa, Olivia Kituuka, Dan Namuguzi, Cynthia Ndikuno, Samuel Kirunda, David Mukunya, Moses Galukande

**Affiliations:** Department of Surgery, College of Health Sciences, Makerere University, Mulago Hill road, P.O Box 7072, Kampala, Uganda East Africa; Department of Nursing, College of Health Sciences, Makerere University, Kampala, Uganda; Department of Paediatrics, College of Health Sciences, Makerere University, Kampala, Uganda; Department of Surgery, School of Medicine, College of Health Sciences, Makerere University, P.O Box 7072 Kampala, Uganda

**Keywords:** Intra-abdominal pressure, Intra-abdominal hypertension, Emergency laparotomy, Mortality

## Abstract

**Background:**

Intra-abdominal hypertension (IAH) is defined as a sustained elevation in intra-abdominal pressure (IAP) greater than or equal to 12 mmHg. IAH has been shown to cause organ derangements and dysfunction in the body. Objective screening of IAH is neither done early enough nor at all thus leading to significant morbidity and mortality among surgical patients. The epidemiology and outcome of IAH among surgical patients has not been documented in Uganda. The aim of this study was to determine the prevalence, incidence and outcome of intra-abdominal hypertension among patients undergoing emergency laparotomy.

**Methodology:**

Prospective observational study, conducted from January to April 2015 among patients undergoing emergency laparotomy. Inclusion criteria was; age >7 yrs, scheduled for emergency laparotomy, able to lie supine. Exclusion Criteria: pregnant, failed urethral catheterization, known cardiac, renal and respiratory disorders. Consecutive sampling was used. IAP, blood pressure, heart rate, respiratory rate, Sp02, Serum creatinine, Serum urea, and Urine output were measured preoperatively and postoperatively at 0, 6, 24 and 48 h. IAH was defined as IAP > 12 mmHg on three consecutive readings 3 min apart.

**Results:**

In total 192 patients were enrolled. Mean age ± SD was 14.25 (±3.16) yrs in the paediatrics and 34.4(±13.72) yrs in the adults with male preponderance 65 and 80.7 % respectively. The prevalence of IAH was 25 % paediatrics and 17.4 % adults and the cumulative incidence after surgery was 20 % paediatrics and 21 % adults. In paediatrics, IAH was associated with mortality at 0 h postoperatively, RRR = 1:24, 95 % CI (1.371–560.178), *p*-value 0.048. In adults, the statistically significant outcomes associated with IAH were respiratory system dysfunction RRR1:2.783, *p*-value 0.023, 95 % CI (1.148–6.744) preoperatively and mortality RRR 1:2.933, *p*-value 0.034, 95 % CI (1.017–8.464) at 6 h, RRR 1:3.769, *p*-value 0.033, 95 % CI (1.113–12.760) at 24 h postoperatively.

**Conclusion:**

The prevalence and incidence of IAH in the paediatrics and adults group in our study population were high. IAH was associated with mortality in both adult and paediatrics groups and respiratory system dysfunction in adult group. This calls for objective monitoring of intraabdominal pressure in patients undergoing emergency laparotomy with the aim of reducing associated mortality.

## Background

Intra-abdominal hypertension is a sustained or repeated elevation in Intra-Abdominal pressure (IAP) greater than or equal to 12 mmHg [1]. IAP is the steady-state pressure concealed within the abdominal cavity [1]. It‘s usually increased in abdominal surgical emergencies. This increased IAP leads to significant organ dysfunction; respiratory, cardiac, renal, gastrointestinal which inevitably leads to increase morbidity and mortality [2–5].

The elevated Intra abdominal pressure leads to a splinting effect on the diaphragm resulting in reduction in lung and chest wall compliance hence the respiratory dysfunction [6, 7]. When the IAP is ≥ 15 mmHg; there is impairment of venous return from the inferior vena cava. At about ≥20 mmHg, there is substantial collapse of abdominal veins (mesenteric, renal and inferior venacava, etc.) hence the significant drop in venous return and this will lead to reduced cardiac output [7–9]. The second effect on the heart is the elevated after load due to increased systemic vascular resistance mainly from the high intra-abdominal pressure and increased intrathoracic pressure [8]. The reduced after load in addition to the venous compression will in turn affect the central nervous system, renal and gastrointestinal system through ischaemia [7, 9].

Despite the growing awareness of the IAH and its adverse consequences; this has coincided with a significant increase in the number of publications related to the theme, many clinicians are not able to recognise or diagnose it early [10, 11] and it’s currently not a routine practice to measure IAP in Uganda.

Therefore in this study we aimed to determine the prevalence, incidence and outcome of IAH among patients undergoing emergency laparotomy.

## Methods

### Study design

A prospective observational study.

### Study setting

This study was conducted in the surgery department of Mulago National Referral Hospital (MNRH), Kampala district. MNRH is Uganda’s national referral hospital. It is also the teaching hospital for the Makerere University College of Health Sciences. It has a total bed capacity of 1500, an inpatient turnover of 120,000 patients and attends to over 480,000 outpatients annually. The study was conducted between January and April 2015. Patients were followed up until discharge or death.

### Participants

All patients above 7 yr that were scheduled for emergency laparotomy in the surgery department of MNRH were eligible for the study provided they were not pregnant, urinary catheterization was possible and did not have any of the following; pelvic fractures, haematuria, neurogenic bladder, cardiac, renal or respiratory conditions.

### Study procedure, measurements and sample collection procedure

A patient was included in the study only after a decision to operate upon him/her was taken by the attending surgical team. Patient particulars (age, sex, tribe, weight, height, past medical history) were noted along with the diagnosis and indication for surgery. Measurement of IAP was taken three times on each occasion and the average was recorded. The measurements were done in the preoperative period and then postoperatively at 0, 6, 24 and 48 h. Zero hour was taken as time of up to 3 h after surgery. If IAP remained below 12 mmHg, measurements were discontinued after 24 h. Other parameters were measured preoperatively and then after surgery at 0, 6, 24 and 48 h. These included; blood pressure, pulse rate, respiratory rate, oxygen saturation (SpO2), temperature, urine output, Glasgow coma score, duration of surgery, blood urea, serum creatinine. Zero hour was taken as time of up to 3 h after surgery.

Post-operational findings to note include: duration of hospital stay, morbidity (burst abdomen), and mortality. The patients were followed up until discharge for morbidity (burst abdomen, relaparotomy, wound dehiscence and wound sepsis) and mortality.

### Details of the measurement of the IAP

The patient was placed in supine position and catheterized with a Foley’s catheter. The bladder was drained until no urine was flowing out from the catheter. The Unometer (UnoMeter™ Abdo-Pressure™ manufactured Unomedical, Uno label 005–2) was connected to the Foley’s Catheter then 20 mls (10–20 mls in Paediatrics) of sterile saline was infused into the bladder through an opening on the Unometer (as recommended by the manufacturer of the Unometer). The tubing of the collecting bag was clamped. The symphysis pubis was the point of zero reference. The patient was told to expire and hold their breath, in that moment the pressure was measured in millimeters of mercury. The measurement was repeated three times with a gap of about 3 min between the readings.

### Study variables

#### Independent variables

Patient’s age (in complete years), gender, weight (in kilograms), height (in meters), diagnosis, duration of surgery (in minutes).

#### Dependent variables

Renal dysfunction(creatinine, urea and urine output), respiratory dysfunction(Respiratory rate, SpO_2_, cardiovascular dysfunction(MAP, Heart rate), central nervous system dysfunction(GCS), intra-abdominal pressure duration of hospital stay, morbidity (burst abdomen, relaparotomy, wound dehiscence and wound sepsis), and mortality.

#### Interpretation of findings

Grading of intra-abdominal hypertension for adults

1. Grade I: 12–15 mmHg;
2. Grade II: 16–20 mmHg;
3. Grade III: 21–25 mmHg; and
4. Grade IV: >25 mm Hg

Grading of intra-abdominal hypertension for paediatrics

1. Grade I: 10–12 mmHg,
2. Grade II: 13–15 mmHg,
3. Grade III: 16–19 mmHg; and
4. Grade IV: ≥ 20 mmHg

Patients (adults) were considered to have ACS when they had IAH > 25 mmHg with evidence of a newly developed organ dysfunction. In Paediatric group, ACS was sustained IAP of greater than 10 mmHg associated with new organ dysfunction/failure. Intervention was left for the attending surgeon to decide.

### Organ system derangement

Cardiovascular system

1. MAP < 60 mmHg
2. Heart rate > 100/min
3. Both of the above

Respiratory system

1. Respiratory rate > 20/min or
2. SpO2 < 90 % or
3. Patient in need of ventilatory support or
4. Any two or all of the above

Renal system

1. Serum creatinine > 106 umol/L or
2. Blood urea > 6.4 umol/L or
3. Urine output < 25 ml/h or
4. Any two or all of the above

Central nervous system

Glasgow Coma Scale: 15–14; Mild, 13–9; Moderate, <8; Severe.

Below 14//15, was be considered as CNS dysfunction

### Study size

The sample size for prevalence and incidence was calculated using the modified Keish and Leslie formula using an estimated proportion of 50.5 %, from a multicentre epidemiological study [12], and a precision of 0.05. The estimated sample size for comparing IAH and outcomes was derived from a general formula for calculating the total sample size using the z statistic for comparison of two proportions with an estimated proportion of 80 % from an Indian study [13]. The larger sample size of 141 patients was considered.

### Statistical methods

#### Data entry

Data was double entered into Epi Data version 3.1 with range, consistency and validity checks embedded to ensure accuracy of data. The data was stored on a computer hard drive that is password protected to ensure confidentiality and backed up on separate external hard drives kept in separate locations.

#### Data analysis

Univariate analysis was performed for baseline factors of the study. For continuous variables such as age; means (standard deviations) and median (interquartile range) if data was skewed, were reported. For categorical variables, proportions and percentages were reported and findings displayed in frequency distribution tables.

Multivariate analysis using the multinomial logistic regression at 95 % confidence interval and *P*-value <0.05 statistical significance, organ dysfunction, morbidity rate, mortality rate were considered as outcomes of IAH. Results were reported in relative risk ratios and *p*-value.

#### Quality control

The recruited research assistants were trained on the use of the data collection tool, how to do the different measurements and patient approach. The data collection tool was pretested. We cross-checked the data daily to ensure completeness with double data entry. An accurate history and physical examination of the patients was done.

Measurements of the IAP were done three times 3 min apart and the average was recorded and to increase accuracy. Standardized machines were used to measure other parameters such as SpO2, MAP, and PR etc.

Data cleaning and entry was done occasionally. There was periodic data evaluation. All questionnaires were safely stored to enable reference in case of data loss.

### Ethical consideration

Informed written consent was obtained from the participants; a translated consent form was availed to non-English speaking respondents. Accent was obtained from the children and written informed consent from the guardian. Confidentiality was observed through strict storage of data and no use of names. Ethical Approval was obtained from; Mulago Hospital ethics committee, Makerere University school of medicine ethics and research committee and the Uganda National council science and technology.

## Results

### Characteristics of the study participants

A total of 192 patients were enrolled in this study. Of these, in the paediatric group 13 (65.5 %) patients and in adult group 138 (80.7 %) were male. Patients’ age ranged from 9–86 years, paediatrics (9–18) years and adults (19–86) years. The paediatric mean age was 14.25 years (SD ± 3.16) and adult age was 34.4 yrs (SD ± 13.72). The paediatric age median was 15 years and adult median 30 years (See Table 1).

**Table 1:** A table showing descriptive characteristics of the study participants

Characteristic	Paediatric		Adults	
	Frequency(*N* = 20)	Proportion (%)	Frequency(*N* = 172)	Proportion (%)
Age				
Mean (SD)	14.25(±3.16)		34.46(±13.72)	
Median	15.0		30.0	
Gender				
Male	13	65.5	138	80.7
BMI				
Underweight (<18.5)	10	50.0	14	8.1
Normal weight (18.5–24.9)	10	50.0	132	76.7
Overweight (25.0–29.9)	−	0.0	14	8.1
Class I obesity (30.0–34.9)	−	0.0	5	2.9
Class II obesity (35.0–39.9)	−	0.0	2	1.2
Referral				
Yes	12	60.0	93	54.4
Indication for emergency laparotomy				
Intra-abdominal sepsis	14	70.0	113	66.1
Intestinal obstruction	5	25.0	11	6.4
Trauma	1	5.0	43	25.1
Others^1^	0	0.0	4	2.3
Operative findings				
Trauma related	4	20.0	10	5.8
Non trauma relate	15	75.0	144	84.2
Duration of surgery				
<60 min	4	20	48	27.9
60 min–<90 min	9	45.0	50	29.1
90 min–<2 hours	6	30.0	40	23.3
>2 hours	0	0.0	15	8.7
Morbidity				
Present	4	20.0	42	24.4
Mortality				
Death occurred	4	20.0	42	24.4

Paediatric IAH ≥10 mmHg and Adult IAH ≥ 12 mmHg

### Intra-abdominal hypertension

IAH was reported as No IAH for values (<10 mmHg) paediatrics, (<12 mmHg) adult or IAH for values (≥10 mmHg) paediatrics and (≥12 mmHg) adults. Table 2 shows the different percentages of the patients who presented with or developed IAH and those who did not develop IAH both preoperatively and postoperatively.

**Table 2:** ATable showing prevalence and incidence of IAH before and after emergency laparotomy

Characteristic	Paediatric		Adults	
	Frequency(*n* = 20)	Proportion (%)	Frequency (*n* = 172)	Proportion (%)
Preoperative				
No IAH	13	65.0	134	77.9
IAH	5	25.0	30	17.4
Overall Postoperative				
No IAH	15	75.0	110	63.9
IAH	4	20.0	37	21.5
IAH at 0 hours (*n* = 153)				
No	14	70.0	122	70.9
Yes	3	15.0	14	8.1
IAH at 6 hours (*n* = 148)				
No	18	90.0	108	62.8
Yes	0	0.0	22	12.8
IAH at 24 hours (*n* = 148)				
No	15	75.0	117	68.1
Yes	1	5.0	15	8.7
IAH at 48 hours (*n* = 83)				
No	5	10.0	63	36.6
Yes	0	0.0	15	8.7

### Prevalence and incidence of IAH

The prevalence of IAH in this study was 25 % paediatrics and 17.4 % adults. Overall incidence of IAH was 20 % paediatrics and 21 % adults. The incidence of IAH at 0, 6, 24 and 48 h is presented in the Table 2.

### Outcomes associated with IAH among patients scheduled for emergency laparotomy

Multivariate analysis was carried out using the multinomial logistic regression in order to determine the significant outcome factors for IAH. Tables 3, 4, 5, and 6 below show IAH as a predictor of the outcomes of organ dysfunction (respiratory and renal), morbidity and mortality in paediatrics and adults groups.

**Table 3:** Multivariate analysis showing IAH as a predictor of respiratory dysfunction and renal dysfunction among adult patients scheduled and underwent emergency laparotomy

Variable	RRR	95 % CI	*P*-value
Respiratory dysfunction			
IAH preoperative			
No IAH	1		
IAH	2.783	1.148–6.744	**0.023**
IAH Postoperative			
No IAH	1		
IAH	1.399	0.661–2.962	0.379
IAH Postop 0 hr			
No IAH	1		
IAH	0.873	0.285–2.669	0.811
IAH Postop 6 hrs			
No IAH	1		
IAH	0.952	0.379–2.396	0.917
IAH Postop 24 hrs			
No IAH	1		
IAH	1.989	0.664–5.963	0.219
IAH Postop 48 hrs			
No IAH	1		
IAH	0.606	0.193–1.905	0.391
Renal Dysfunction			
IAH preoperative			
No IAH	1		
IAH	0.538	0.201–1.439	0.217
IAH Postoperative			
No IAH	1		
IAH	0.522	0.218–1.255	0.147
IAH Postop 0 hr			
No IAH	1		
IAH	1.139	0.358–3.621	0.826
IAH Postop 6 hrs			
No IAH	1		
IAH	0.588	0.201–1.722	0.333
IAH Postop 24 hrs			
No IAH	1		
IAH	0.138	0.017–1.084	0.060
IAH Postop 48 hrs			
No IAH	1		
IAH	0.331	0.068–1.606	0.170

Highlighted variables statistically significant findings since *p*-value < 0.05

**Table 4:** Multivariate analysis showing IAH as a predictor of respiratory dysfunction and renal dysfunction among Pediatric patients scheduled and underwent Emergency laparotomy

Variable	RRR	95 % CI	*P*-value
Respiratory dysfunction			
IAH preoperative			
No IAH	1		
IAH	3.333	0.319–34.829	0.315
IAH Postoperative			
No IAH	1		
IAH	6.500	0.555–76.175	0.136
IAH Postop 0 hr			
No IAH	1		
IAH	3.000	0.177–50.784	0.447
IAH Postop 6 hrs			
No IAH	1		
IAH	1	−	−
IAH Postop 24 hrs			
No IAH	1		
IAH	1.900*10^8^	0	0.998
IAH Postop 48 hrs			
No IAH			
IAH			
Renal Dysfunction			
IAH preoperatively			
No IAH	1		
IAH	6.31*10^8^	0	0.998
IAH Post-operatively			
No IAH	1		
IAH	1.5	0.164–13.749	0.720
IAH 0 hrs Postop			
No IAH	1		
IAH	2.667	0.193–36.756	0.464
IAH 6 hrs Postop			
No IAH	1		
IAH	1		
IAH 24 hrs Postop			
No IAH	1		
IAH	3.35*10^7^	0	0.994
IAH 48 hrs Postop			
No IAH			
IAH			

Highlighted variables statistically significant at *p*-value < 0.05

**Table 5:** Multivariate analysis showing IAH as a predictor of morbidity rate and mortality among adults patients scheduled and underwent Emergency laparotomy

Characteristic	RRR	95 % CI	*P*-value
Morbidity Rate			
IAH preoperatively			
No IAH	1		
IAH	1.955	0.844–4.529	0.118
IAH Postoperatively			
No IAH	1		
IAH	1.078	0.476–2.444	0.857
IAH 0 hrs Postop			
No IAH	1		
IAH	0.398	0.085–1.869	0.243
IAH 6 hrs Postop			
No IAH	1		
IAH	0.668	0.227–1.964	0.464
IAH 24 hrs Postop			
No IAH	1		
IAH	1.125	0.359–3.528	0.840
IAH 48 hrs Postop			
No IAH	1		
IAH	1.433	0.449–4.578	0.543
Mortality Rate			
IAH preoperatively			
No IAH	1		
IAH	2.249	0.918–5.506	0.076
IAH Postoperatively			
No IAH	1		
IAH	1.043	0.399–2.729	0.930
IAH 0 hrs Postop			
No IAH	1		
IAH	1.829	0.523–6.395	0.345
IAH 6 hrs Postop			
No IAH	1		
IAH	2.933	1.017–8.464	**0.047**
IAH 24 hrs Postop			
No IAH	1		
IAH	3.769	1.113–12.760	**0.033**
IAH 48 hrs Postop			
No IAH	1		
IAH	0.338	0.040–2.843	0.318

Highlighted variables statistically significant at *p*-value < 0.05

**Table 6:** IAH as a predictor of morbidity rate and mortality among Pediatric patients scheduled and underwent Emergency laparotomy

Characteristic	RRR	95 % CI	*P*-value
Morbidity rate			
IAH preoperative			
IAH	1		
No IAH	1.25	0.087–17.975	0.870
IAH Postoperative			
IAH	1		
No IAH	1.333	0.099–17.823	0.828
IAH Postop 0 hr			
IAH	1		
No IAH	3.67*10^8^	−	0.998
IAH Postop 6 hrs			
IAH	1		
No IAH		−	−
IAH Postop 24 hrs			
IAH	1		
No IAH	1.900*10^8^		0.998
IAH Postop 48 hrs			
IAH			
No IAH			
Mortality Rate			
IAH preoperative			
No IAH	1		
IAH	5.39*10^9^	−	0.999
IAH Postoperative			
No IAH	1		
IAH	13	0.771–219.107	0.075
IAH Post op 0 hr			
No IAH	1		
IAH	24	1.028–560.178	**0.048**
IAH Post op 6 hrs			
No IAH			
IAH			
IAH Post op 24 hrs			
No IAH	1		
IAH	7.51*10^7^	−	0.997
IAH Post op 48 hrs			
No IAH	1		
IAH	0.339	0.041–2.833	0.318

Highlighted variables statistically significant at *p*-value < 0.05

In the paediatrics group, Patients with IAH postoperatively at 6 h were more than twenty four times at risk of dying as compared to those without IAH, RRR = 1:24, 95 % CI (1.371–560.178), *p*-value 0.048.

In the adults group, patients with IAH preoperatively were 2.7 times more likely to develop respiratory dysfunction compared to those without IAH, RRR = 1:2.783, 95 % CI (1.148–6.744), and *p*-value 0.023.

Patients with IAH postoperatively at 6 h and at 24 h were noted to be more than 2.933 and 3.769 times more likely to die than those without IAH, 95 % CIs (1.017–8.464) (1.113–12.760) and *p*-values 0.047, 0.033 respectively

There was an increasing likelihood or risk of dying with increasing time (hrs) among patients postoperatively with IAH.

## Discussion

This study was conducted to find out the epidemiology of IAH and the association of IAH with organ dysfunction, morbidity (e.g. burst abdomen, re-laparotomy) and mortality among surgical patients undergoing emergency laparotomy. We found the prevalence to be 25 % paediatrics and 17.4 % adults, and incidence to be 20 % paediatrics and 21 % adults. IAH is a significant predictor of mortality and respiratory dysfunction.

### Basic Characteristics of the study participants

In both the paediatrics and adults groups, there was a male predominance that is 65 % in paediatrics and 80.7 % in adults; a similarity is seen in a number of studies. A study done in India had 76 % males [13], a study in children 57 % male [14], in USA two studies were done which reflected males 70 % [15] and 72 % [16]. The mean age in the paediatrics group was 14.25 years (SD ± 3.16) and adult age was 34.4 yrs (SD ± 13.72). The median age for paediatric and adults group was 15 and 30 years respectively which is consistent with the Uganda population statistics which show that Uganda has a young population [17]. In this study, intra-abdominal sepsis had 66 % of the study population, which is similar with findings from Khan et al. (2010) where 64 % presented with intra-abdominal sepsis.

### Intra-abdominal pressures

The mean IAP before and after emergency laparotomy were 15.4 (SD ± 3.6) mmHg and 14.21 (SD ± 5.0) mmHg, respectively. The mean IAP in the study group of Khan et al. before and after decompressions were 16.6 (SD ± 9.4) mmHg and 10.3 (SD ± 3.1) mmHg, respectively [13]. Meldrum et al. reported higher values of preoperative IAP, 27 (SD ± 2.3) [15]. This can be explained by the observation that in our study, 66 % of the patients had peritonitis secondary to gut perforation leading to elevated IAP which, after decompression and removal of liters of fluids and gas, returned to normal level immediately.

### Prevalence of IAH

The prevalence of IAH in our study was 25 % paediatrics and 17.4 % adults. In the adults group, this was slightly lower than Murtaza et al. who showed a prevalence of 28 % [18], however this can be explained by the fact that Murtaza et al. looked at ICU patients who are generally sicker than the general patients we looked at. Again in the paediatrics group, the prevalence was lower compared to Ozden et al. with 49.3 %, this is still explained by the different study populations, and this study was carried out in the emergency ward setting compared to NICU or PICU.

### Incidence of IAH

In the adult group, the incidence of IAH in literature varies from 2 to 81 % depending on the values used to defined IAH and patient population [19, 20]. However, only a few studies have reported the incidence in paediatrics, 12.6 % Thabet et al. [21], and 9 % Divarci et al. [14]. The incidence of IAH in our study was 20 % paediatrics and 21 % adults. The incidence in the adult group at the 0, 6, 24 and 48 h postoperatively was 8.1, 12.8, 8.7 and 8.7 % respectively. At 6 h the incidence of IAH increases and then drop at 24 and 48 h, this is similar to Khan et al. reported 0 % at 0 h, 3.55 % at 6 h and 0 % at 24 h [13]. The incidence of 21 % in the adults group is high therefore close attention should be paid to these patients postoperatively to avoid poor outcome. The incidence in paediatrics group at 0, 6, 24 and 48 h was 15, 0, 5 and 0 %.

### Outcome factors associated with IAH

In the adults group, the statistically significant outcomes associated with IAH include mortality and respiratory system dysfunction. Preoperative IAH was significantly associated with respiratory system dysfunction while post-operative IAH was significantly associated with mortality. Khan et a[13]l reported similar findings and the preoperative respiratory dysfunction is explained by the splintage of the diaphragm as the IAP progressively raises, however the after surgery respiratory function could have improved due to the decompression from surgery.

The mortality associated with IAH postoperatively at 6 and 24 h shows at need to objectively measure IAP in patients at risk hence early recognition and proceeds with effective intervention.

In the paediatrics group, the significant outcome associated with IAH after multivariate analysis was mortality at 0 h, which could be explained by probable delay in surgical intervention or recognition and appropriate treatment of IAH.

### Limitation of the study

The measurements may have varied depending on whether if they were taken at end of inspiration or at the end of expiration which may have led to under estimation of the readings. Sessions were conducted to train the research assistants to correctly measure the IAP.

IAP measurements were between 6 h interval; hence we may have missed episodes of IAH between measurements.

At IAP measured omitted at 48 h if pt has no episode of IAH, pre-op and post-op up to 24 h. This was done for the concern of causing urinary tract infections in the patients, hence the reading 48 h was to be assumed as normal (but this was not analysed) hence our few numbers at 48 h.

## Conclusion

The prevalence and incidence of IAH in both paediatrics and adult groups in this study were high. IAH was significantly associated with mortality postoperatively and respiratory dysfunction preoperatively in the adult group but only mortality postoperatively in the paediatrics group. These conclusions add emphasis to the need to objectively monitor IAP in these patients undergoing emergency laparotomy so as to recognise and treatment IAH early.

### Recommendations

Objective routine IAP measurements should be considered for all patients undergoing emergency laparotomy. Clinical protocols should be designed to guide the management of patients with IAH in the accidents and emergency surgery wards. Resources should also be channeled towards procurement of equipment required to monitor the IAP in these patients. More studies are needed to demonstrate proper guidelines on monitoring of IAP in patients undergoing emergency laparotomy.
